# Application of Hydrazine-Embedded Heterocyclic Compounds to High Voltage Rechargeable Lithium Organic Batteries

**DOI:** 10.1038/s41598-017-19037-8

**Published:** 2018-01-12

**Authors:** Takeshi Shimizu, Koji Yamamoto, Palash Pandit, Hirofumi Yoshikawa, Shuhei Higashibayashi

**Affiliations:** 1 School of Science and Technology, Kwansei Gakuin University, 2-1 Gakuen, Sanda, Hyogo 669-1337 Japan; 2 Institute for Molecular Science, Myodaiji, Okazaki 444-8787 Japan; 3 Present Address: Faculty of Pharmacy, Keio University, 1-5-30 Shibakoen, Minato-ku, Tokyo, 105-8512 Japan

## Abstract

Hydrazine-embedded heterocyclic compounds with dimeric dimethylacridine (**1b**), carbazole (**2b**), and phenothiazine (**3b**) skeletons were applied to cathode active materials of rechargeable lithium organic batteries, and the performance of the batteries was evaluated. The charge/discharge curves exhibited clear plateaus in the high voltage range of 3.3–3.7 V. The capacities of the plateau regions were comparable to the calculated capacities corresponding to the one-electron redox of the molecules. The amount of the active compound **3b** could be increased up to 30 wt% in the electrode composite, and fast charge/discharge performance was also observed.

## Introduction

**Figure 1:**
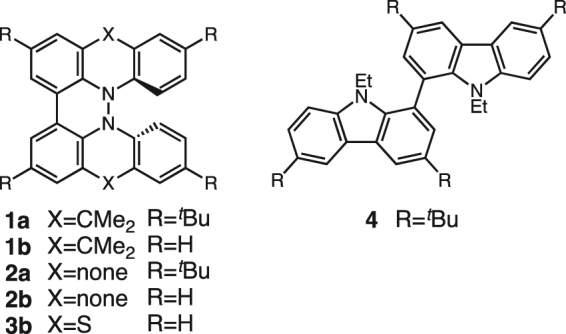
Hydrazine-embedded polyheterocyclic compounds **1**–**3** and reference compound **4**.

Research examples of polyheterocyclic compounds possessing an embedded hydrazine structure in the π-conjugated skeletons are very limited, even though hydrazine units are well-known functional groups^1–6^. In recent years, we have reported novel hydrazine-embedded polyheterocyclic compounds including hydrazinohelicenes **1** and **2** (Fig. 1) and demonstrated that these compounds exhibit characteristic chemical and physical properties such as acid-responsive reversible electron transfer disproportionation, reversible two-electron oxidation over a wide potential range, highly stable radical cations, long-wavelength absorption and emission, and redox-dependent transformation of the geometry owing to the existence of the embedded hydrazine structure^7–11^. Rechargeable organic batteries have received much attention and many organic materials have been applied as the cathode active materials^12–15^. However, hydrazine-containing compounds have not been used as an active material of a rechargeable organic battery to date. The excellent reversible redox properties of hydrazine-embedded polyheterocyclic compounds prompted us to use these compounds as cathode materials of a rechargeable Li-ion organic battery. Here, we report the performances of rechargeable lithium organic batteries using hydrazine-embedded compounds **1b**-**3b** with dimeric dimethylacridine, carbazole, and phenothiazine skeletons as well as that of a non-hydrazine-embedded carbazole dimer **4** as a reference compound.

## Results and Discussion

**Figure 2:**
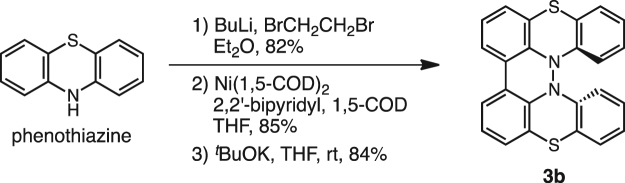
Synthesis of **3b**.

Unsubstituted **1b**-**3b** were chosen as cathode active materials rather than *t*-butyl-substituted **1a** and **2a**, since **1b** and **2b** had lower solubility in the electrolyte of the battery [LiPF_6_ in ethylene carbonate/diethylcarbonate (1:1)] than **1a** and **2a**^7–9^. Low solubility of the active materials in the electrolyte of a battery is generally desired for maintaining the capacity and recycle performance of the battery. Compounds **1b** and **2b** were prepared in 8 steps from *N*-phenylanthranilic acid and in 5 steps from carbazole according to our previous reports, respectively^7–9^. Phenothiazine dimer **3b**^6^ was synthesized by bromination of phenothiazine, Ni-mediated coupling of bromophenothiazine, and aerobic oxidation under basic conditions in 3 steps (Fig. 2). Compound **4** was prepared according to a literature procedure^16^.

**Figure 3:**
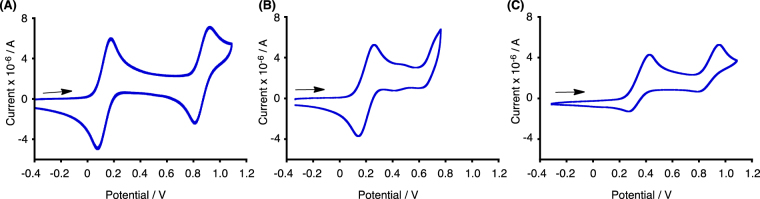
Cyclic voltammograms of (**A**) **1b**, (**B**) **2b**, and (**C**) **3b** (Pt electrode, 0.1 M Bu_4_NClO_4_ in CH_2_Cl_2_, vs. Fc/Fc^+^).

**Figure 4:**
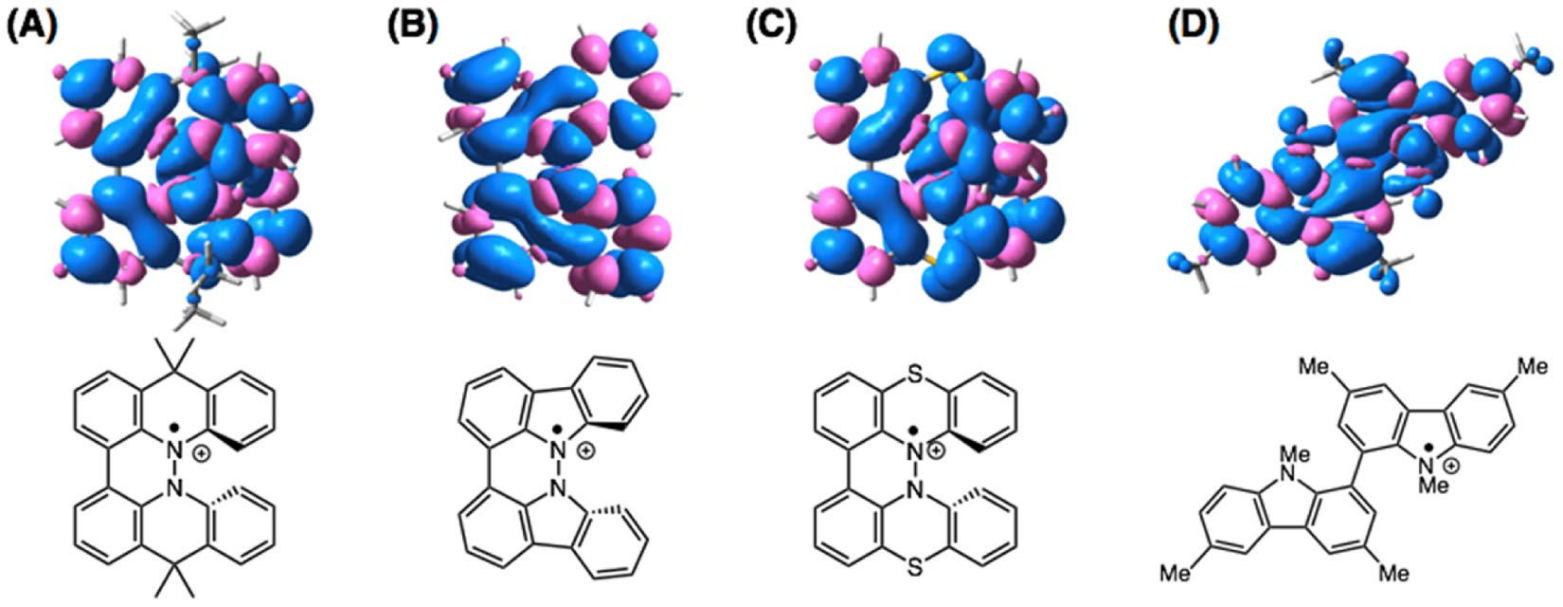
Calculated spin density distributions of monocation radicals of **1b**-**3b** and **4** [UωB97XD/6–31 G(d)]. Blue and pink colors indicate positive and negative spin density, respectively. For **4**, *t*-butyl and ethyl groups were replaced by methyl groups.

**Figure 5:**
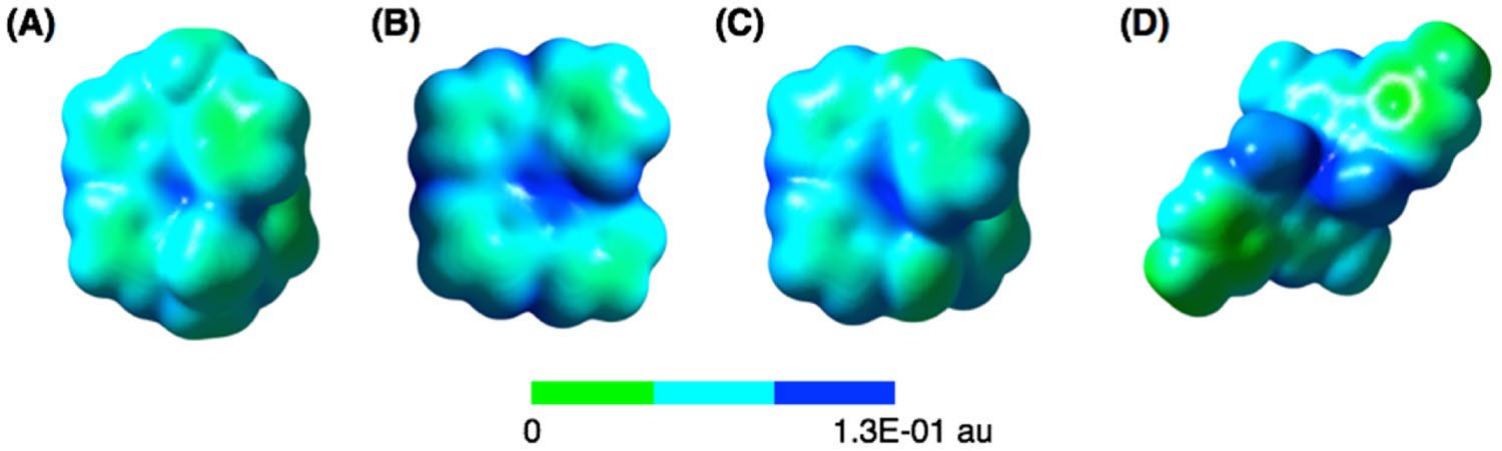
Calculated electrostatic potential (ESP) maps (isosurface at 0.02 au) of monocation radicals of **1b**-**3b** and **4** [UωB97XD/6-31 G(d)].

The electrochemical oxidation potentials of **1b**-**3b** were determined by cyclic voltammetry (CV) measurements (Fig. 3, Table 1). Dimethylacridine dimer **1b** exhibited reversible two-step oxidations at 0.13 V and 0.87 V^8^. Carbazole dimer **2b** showed a reversible one-electron oxidation at 0.36 V, while the second oxidation was irreversible. Phenothiazine dimer **3b** exhibited two-step oxidations at 0.34 V and 0.83 V^6^, while the second oxidation was quasi-reversible. Carbazole dimer **4** without a hydrazine unit was reported to exhibit reversible two-step oxidations at 0.70 V and 0.93 V^16^. Judging from the first reversible one-electron oxidation waves of **1b-3b** and **4**, the monocation radicals of **1b-3b** and **4** are stable under the electrolysis conditions, which suggested that their one-electron redox properties would be applicable to organic rechargeable batteries. DFT calculations [UωB97XD/6–31 G(d)] of the monocation radical of **1b**-**3b** and **4** were conducted to obtain information about the electronic states of the monocation radical species. The calculated spin density distributions and the electrostatic potential (ESP) maps are shown in Figs 4 and 5, respectively. For the calculation of **4**, *t*-butyl and ethyl groups were replaced with methyl groups. The calculated spin densities of all the monocation radicals of **1b**-**3b** and **4** are delocalized over the entire skeleton, leading to the stability of the monocation radicals. The calculated ESP maps of **1b**-**3b** show that the inside part of the molecules around the nitrogen atoms is positively charged in addition to the aromatic hydrogen atoms. In contrast, the calculated ESP map of **4** indicated that the alkyl groups on the nitrogen atoms directed to the outside of molecules are positively charged.

**Table 1:** Oxidation Potentials (vs. Fc/Fc^+^).

Compounds	*E*^1^/V	*E*^2^/V
**1b**	0.13	0.87
**2b**	0.36	—
**3b**	0.34	0.83
**4** ^16^	0.70	0.93

**Figure 6:**
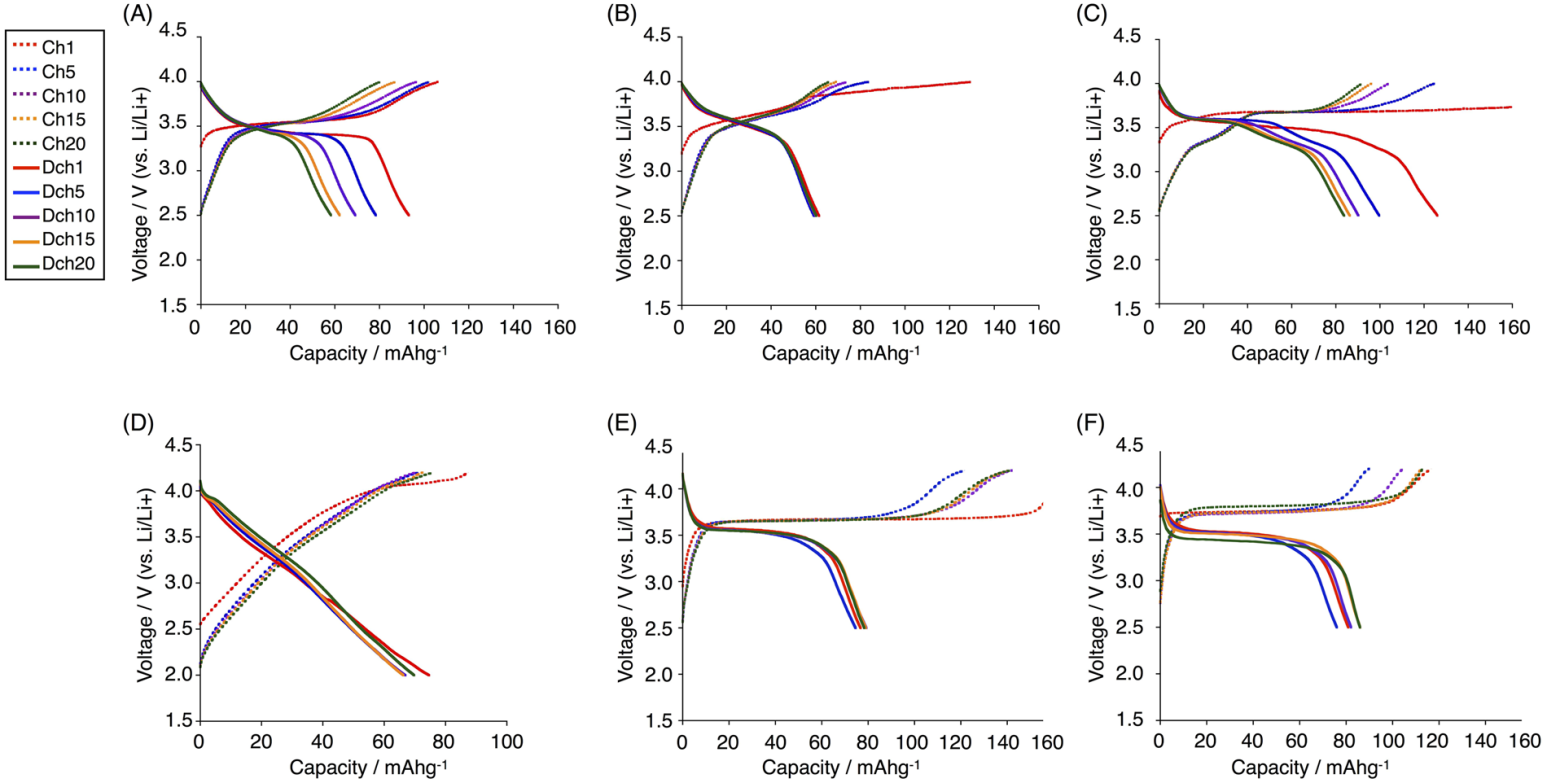
Selected charge/discharge curves of fabricated batteries using (**A**) **1b** (rate = 10.4 mAg^−1^), (**B**) **2b** (rate = 14.9 mAg^−1^), (**C**) **3b** (rate = 19.2 mAg^−1^), (**D**) **4** (rate = 57.3 mAg^−1^) by method A and those using (**E**) **3b** (rate = 200 mAg^−1^), (**F**) **3b** (rate = 500 mAg^−1^) by method B. The potential is shown vs. Li/Li^+^ and the capacity is normalized by the weight of the compounds.

**Figure 7:**
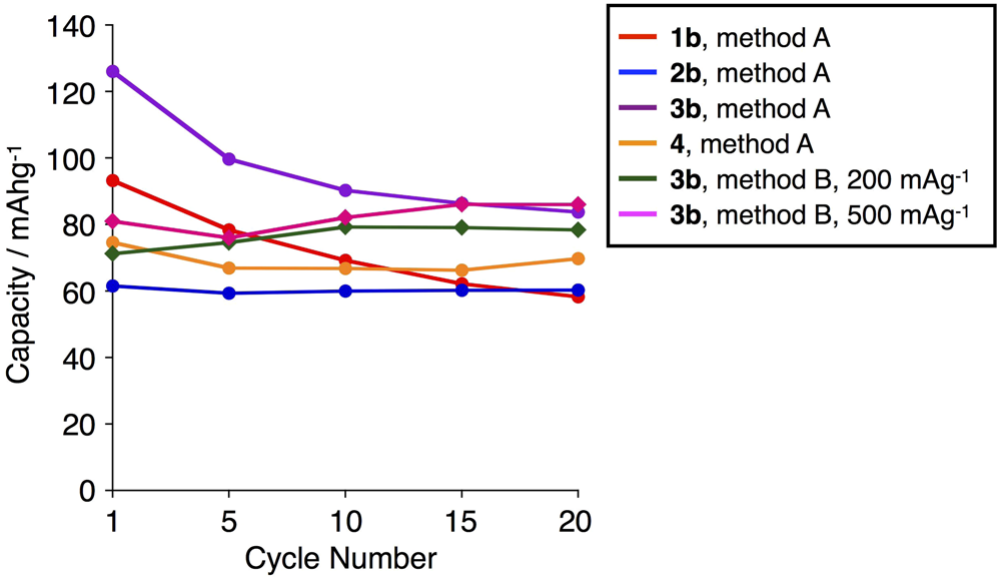
Cycle performance of discharged capacities at 2.5 V (**1b**-**3b**) or 2.0 V (**4**).

We then applied these compounds as cathode-active materials for Li-ion batteries. Cathode electrode composites (0.5 mm thickness) were prepared by mixing the compound (10 wt%), polyvinylidene difluoride (PVDF) (20 wt%), and conductive carbon black (TOKA BLACK 5500 of TOKAI CARBON) (70 wt%) with *N*-methyl-2-pyrrolidone (Method A). A lithium organic battery was assembled with a Li metal foil anode and the prepared cathode with a porous polymer film separator in an electrolyte solution composed of 1 M LiPF_6_ in ethylene carbonate/diethylcarbonate (1:1) in a coin-shaped cell (2032-type). The charge/discharge performances of the batteries were measured at a constant current [10 mAg^−1^ (**1b**), 15 mAg^−1^ (**2b**), 19 mAg^−1^ (**3b**), 57 mAg^−1^ (**4**)] for 20 cycles between 2.5–4.0 V (**1b-3b**) or 2.0–4.2 V (**4**) on a charge/discharge device (HOKUTO DENKO HJ1010mSM8A). The selected charge/discharge curves and the cycle performance of discharged capacities at 2.5 V (**1b**-**3b**) or 2.0 V (**4**) are shown in Figs 6(A–D) and 7, respectively. The curves of hydrazine-embedded compounds **1b**-**3b** exhibited clear plateaus in the range of high voltage = 3.3–3.7 V in both charge and discharge processes. Compared with the voltages of reported Li-ion organic batteries using representative active materials, the voltages of **1b**-**3b** are higher than those of quinone molecules (typically <3 V) and comparable to those of nitroxide molecules (around 3.6 V)^12–15^. The plateau regions correspond to the chemical one-electron redox processes in the CV, since the obtained capacities are consistent with the calculated ones from one-electron reduction and the one-electron redox voltage in CV (vs. Li^+^/Li) corresponds to the plateau voltage. The voltage of the plateau region of **1b** is lower than that of **2b**, which is consistent with their oxidation potentials (0.13 and 0.36 V for **1b** and **2b**, respectively) in CV. The plateau voltage of **3b** in the discharge process gradually decreased in the range of 3.6–3.2 V, while the plateau for the thin film cathode by using method B was more flat (*vide infra*). The gradual decrease of plateau voltage of **3b** in the pellet cathode is probably due to the higher resistance in the pellet than in the thin film. In contrast to **1b**-**3b**, the curve of **4** did not show a plateau in either charge or discharge processes [Fig. 6(D)]. Since the capacity, which is consistent with the theoretical one, was not decreased significantly by the recycling charge/discharge process, the lack of a plateau for **4** was not due to the decomposition of the compound. This suggests that its redox reaction gradually occurs when the voltage decreases, which is probably caused by the difference in molecular skeleton. The calculated capacities per weight of compounds **1b**-**3b** were 65, 81, and 68 mAhg^−1^ for one-electron redox, respectively. For **3b**, larger capacity was observed in the first discharge process, which is probably due to non-Faradic reaction and SEI in the first discharge process. The observed capacities of **1b** and **3b** were gradually decreased by the cycling charge/discharge process (Fig. 7), but the extent of decrease of capacity per cycle became smaller. In contrast to **1b** and **3b**, the observed capacity of **2b** did not change by the recycling process, which is attributed to the lower solubility of **2b** in the electrolyte solution. The capacity of the plateau region of **3b** was comparable to the calculated one for the one-electron redox, while those of **1b** and **2b** were slightly smaller than the calculated ones. Since phenothiazine dimer **3b** was easily prepared in the fewest steps among **1b**-**3b**, we prepared another cathode electrode composite by a different method B using **3b**. Cathode electrode composites (0.1 mm thickness) were prepared by mixing the compound (30 wt%), polyvinylidene difluoride (PVDF) (10 wt%), and conductive carbon black (TOKA BLACK 5500 of TOKAI CARBON) (60 wt%) with *N*-methyl-2-pyrrolidone on an aluminum sheet. Compared with method A, the amount of **3b** was increased up to 30 wt% and the thickness of the cathode electrode composite was decreased to 0.1 mm. Lithium batteries were assembled with the cathode electrode composite, and the performance was investigated. The charge/discharge performance of the batteries was measured at a constant current (200 mAg^−1^ or 500 mAg^−1^) for 20 cycles between 2.5–4.2 V. Selected charge/discharge curves and the cycle performance of discharged capacities at 2.5 V are shown in Figs 6(E,F) and 7. Compared with the results obtained by using method A [Fig. 6(C)], the plateau region was improved to be more clear. This is probably due to homogeneous electron transfer in the thinner cathode, in which Li ions are quickly intercalated and the conductivity is increased. The capacity was not significantly decreased and the cyclability was also improved (Fig. 7). Based on these results, we increased the current density from 200 mAg^−1^ [Fig. 6(E)] to 500 mAg^−1^ [Fig. 6(F)]. Since the battery performance was nearly maintained, it is assumed that thinner cathodes enabled faster charge of the battery using **3b** even if the active material concentration was high.

## Conclusions

In summary, we have applied hydrazine-embedded heterocyclic compounds **1b**-**3b** with dimethylacridine, carbazole, and phenothiazine skeletons to cathode active materials of rechargeable lithium organic batteries and investigated the performance of the assembled batteries. The charge/discharge curves exhibited clear plateaus in the high voltage range of 3.3–3.7 V. The capacities of the plateau regions were comparable to the calculated capacities corresponding to the one-electron redox of the molecules. The amount of the active compound **3b** could be increased up to 30 wt% in the electrode composite, and fast charge/discharge performance was also observed. This study demonstrated the applicability of hydrazine-embedded heterocyclic compounds to the cathode active materials of a high voltage rechargeable lithium organic battery as a first example. We expect that hydrazine-embedded heterocyclic compounds with excellent reversible redox properties can be applied as a component of a variety of other redox systems including organic electronic devices.

## Methods

### Synthesis of 1,1′-biphenothiazine

1-Bromophenothiazine was prepared according to the literature^17^. A solution of Ni(1,5-COD)_2_ (1.19 g, 4.31 mmol), 2,2′-bipyridyl (673 mg, 4.31 mmol), and 1,5-COD (529 μL, 4.31 mmol) in dry THF (20 mL) was heated at 70 °C under an Ar atmosphere for 10 min. To the solution, 1-bromophenothiazine (1.00 g, 3.40 mmol) in dry THF (10 mL) was added dropwise over 1 min. The solution was stirred at 70 °C for 1 h. The solution was poured in CHCl_3_ and silica gel was added. The suspension was filtered through a silica gel pad, washed by CHCl_3_, and evaporated. The residue was dissolved in CH_2_Cl_2_ (5 mL) and precipitated by addition of hexane (20 mL). The precipitate was filtered to afford 1,1′-biphenothiazine (604 mg, 85% yield) as a colorless solid.

Mp: 250–252 °C. IR (ATR): ν 3364, 2359, 1474, 1425, 1283, 1249, 777, 751, 719, 667 cm^−1^. ^1^H NMR (CDCl_3_): δ 7.08 (2 H, m), 6.99 (2 H, dd, *J* = 18.7, 3.4 Hz), 6.95–6.91 (4 H, m), 6.87 (2 H, ddd, *J* = 19.1, 19.1, 3.8 Hz), 6.80 (ddd, 2 H, *J* = 18.4, 18.4, 3.1 Hz), 6.27 (2 H, dd, *J* = 19.1, 3.1 Hz), 5.78 (2 H, br s) ppm. ^13^C NMR (CDCl_3_): δ 141.0, 139.6, 129.1, 127.4, 127.2, 126.6, 123.0, 122.8, 121.8, 119.8, 118.2, 115.3 ppm. HRMS (EI) (*m*/*z*) for C_24_H_16_N_2_S_2_ (M^+^): calculated 396.0755, found 396.0745.

### Synthesis of 3b

To a solution of potassium *t*-butoxide (323 mg, 2.87 mmol) in THF (10 mL) under air at room temperature was added 1,1’-biphenothiazine (380 mg, 0.958 mmol). The solution was stirred for 4 h. The reaction mixture was quenched by aq. NH_4_Cl at 0 °C and extracted by CH_2_Cl_2_ (20 mL × 3). The combined organic layers were dried over Na_2_SO_4_, filtered through Celite, and evaporated. The residue was dissolved in CH_2_Cl_2_: hexane = 1: 1 and filtered through a silica gel pad. After evaporation of solvent, the residue was suspended in hot MeOH (100 mL) and stored in a fridge overnight. The precipitate was filtered to afford 1,1′,10,10′-biphenothiazine (319 mg, 84% yield) as a yellowish solid. Mp: 183 °C (decomp.). IR (ATR): ν 1468, 1442, 1414, 1220, 1029, 894, 772, 744, 644 cm^−1^. ^1^H NMR (CDCl_3_): δ 7.57 (2 H, dd, *J* = 8.1, 1.1 Hz), 7.43 (2 H, dd, *J* = 7.7, 1.3 Hz), 7.25 (2 H, dd, *J* = 7.8, 1.4 Hz), 7.17 (2 H, dd, *J* = 6.7, 1.2 Hz), 7.14 (2 H, ddd, *J* = 8.2, 7.3, 1.4 Hz), 7.06 (2 H, dd, *J* = 7.7, 7.7 Hz), 6.98 (2 H, ddd, *J* = 7.5, 7.5, 1.3 Hz) ppm. ^13^C NMR (CDCl_3_): δ 146.2, 140.3, 127.9, 127.5, 127.3, 125.3, 124.8, 123.9, 123.4, 121.8, 121.0, 113.7 ppm. HRMS (FAB) (*m*/*z*) for C_24_H_14_N_2_S_2_ (M^+^): calculated 394.0598, found 394.0601.

### Fabrication of batteries

Method A: The cathode-active compound (50 mg), polyvinylidene difluoride (PVDF, Aldrich) (100 mg), and conductive carbon black (TOKA BLACK 5500 of TOKAI CARBON) (350 mg) were mixed using *N*-methyl-2-pyrrolidone (Aldrich). The mixture was spread at a thickness of 0.5 mm onto a polypropylene sheet, cut into a disc with a diameter of 1.6 cm, and dried *in vacuo* after removal of the polypropylene sheet. Under an argon atmosphere, the cathode, a Li metal foil (0.2 mm thickness) (Honjo Metal Co. Ltd.), and a porous polymer film separator (polyolefin) (Celgard 2325, 25 μm thickness) were placed in a coin-shaped cell (2032-type) with an electrolyte solution composed of 1 M LiPF_6_ in ethylene carbonate/diethylcarbonate (1:1) (Kishida Chemicals).

Method B: The cathode-active compound (90 mg), polyvinylidene difluoride (PVDF) (30 mg), and conductive carbon black (180 mg) were mixed using *N*-methyl-2-pyrrolidone. The mixture was spread at a thickness of 0.1 mm onto an aluminum sheet using a doctor blade, dried *in vacuo*, and cut into a disc with a diameter of 1.6 cm. Under an argon atmosphere, the cathode, a Li metal foil (0.2 mm thickness), and a porous polymer film separator (polyolefin) were placed in a coin-shaped cell (2032-type) with an electrolyte solution composed of 1 M LiPF_6_ in ethylene carbonate/diethylcarbonate (1:1).
